# The Development and Initial Validation of the Memorial Symptom Assessment Scale-Long COVID (MSAS-LC): A Promising Tool for Measuring Long COVID

**DOI:** 10.3390/ijerph22050728

**Published:** 2025-05-02

**Authors:** Sadie B. Sommer, Mary S. Dietrich, Julie V. Barroso

**Affiliations:** 1 Center for Research Development and Scholarship, School of Nursing, Vanderbilt University, Nashville, TN 37240, USA; julie.v.barroso@vanderbilt.edu; 2 School of Medicine, Biostatistics, Hearing & Speech, Vanderbilt University, Nashville, TN 37240, USA; mary.dietrich@vanderbilt.edu; 3 School of Nursing, Vanderbilt University, Nashville, TN 37240, USA

**Keywords:** long COVID, symptom assessment, patient-reported outcomes, post-viral conditions, symptom burden, health monitoring, symptom prevalence

## Abstract

Long COVID remains a public health challenge, impacting over 65 million people globally and manifesting as persistent, multisystemic symptoms that complicate both diagnosis and treatment. To address the need for a standardized, patient-centered assessment tool, this study introduces the Memorial Symptom Assessment Scale-Long COVID (MSAS-LC), which evaluates symptom prevalence, frequency, severity, and distress. The MSAS-LC was developed by modifying the Memorial Symptom Assessment Scale to include 45 prevalent Long COVID symptoms. A cross-sectional survey of 261 U.S. adults (129 with Long COVID and 131 without) assessed validity and group differences. Symptom prevalence was analyzed using logistic regression, while symptom burden (frequency, severity, and distress) was compared using generalized linear models. Participants with Long COVID reported significantly higher symptom prevalence and burden across all systems. Memory problems (73.4% vs. 30.5%; OR = 6.29, *p* < 0.001) and post-exertional fatigue (OR = 8.55, *p* < 0.001) were among the most burdensome symptoms. These findings offer preliminary evidence supporting the potential utility of MSAS-LC and underscore the continued public health relevance of individual and collective symptom presentations. The findings suggest the distinct symptom burden, emphasizing the importance of future research to inform diagnostic and treatment strategies. With continued validation, the MSAS-LC may contribute to improved symptom monitoring and care planning in clinical and public health settings.

## 1. Introduction

Long COVID is defined by the World Health Organization (WHO) as the continuation or development of new symptoms after an initial SARS-CoV-2 infection, with symptoms lasting more than two months with no alternative explanation [1]. An estimated 65 million people are struggling with a debilitating multisystem condition that impairs their ability to perform daily activities for several months or years [2], and Long COVID is considered a disability under the Americans with Disabilities Act (section 5.4 and section 1557) [3]. Long COVID is associated with all ages and acute phases of disease severity [4], with the highest percentage of diagnoses observed in individuals ages 50–59 years and those over age 80 [5]. Long COVID can affect individuals regardless of initial disease severity, hospitalization status, or pre-existing conditions, although these factors are known to influence the risk and clinical presentation [6]. Nonetheless, important questions remain about the diagnosis, definitions, disease trajectory, and treatment.

Over 200 symptoms have been associated with Long COVID [7], with some individuals experiencing dozens of symptoms across multiple organ systems [8,9]. Some of the most prevalent symptoms include neurological clustering, such as brain fog and cognitive impairments, and cardiorespiratory clustering, such as breathlessness and fatigue [10,11,12]. The signs and symptoms are so diverse that it has proven challenging to definitively understand the pathological and epidemiological characteristics and ascribe proper terminology to the symptoms. Reviews of Long COVID literature found a lack of uniform symptom terminology, standardized recording methods, and grouping of multiple symptoms under umbrella terms [13,14].

These discrepancies in diagnostic criteria have also led to a wide variation in estimates of prevalence. Estimates in the U.S. range from 5.5% [15] to over 30% [8]. Some argue that these numbers are likely much higher due to many undocumented or misdiagnosed cases. In our fifth year of COVID, we can now observe unabating symptoms for over four years [16]. Nonetheless, the wide variation in prevalence estimates denotes a lack of diagnostic consistency that affects the accuracy and reproducibility of otherwise robust research.

In a clinical setting, the ICD-10-CM code (U09.9, “Post COVID-19 condition, unspecified”) has been applied since October 2021 [17]. However, this single code has proven insufficient in categorizing the phenotype and severity variation seen in Long COVID patients. Considering many long-term COVID symptoms affect individuals’ ability to work and their quality of life [18,19], diagnostic scales have been developed to measure all aspects of health. In the United Kingdom, (SBQ-LC) was analyzed using a Rasch procedure and has 17 scales with 131 items [20], and the modified COVID-19 Yorkshire Rehabilitation Scale measures 22 symptoms on an 11-point numerical rating scale divided into four subscales measuring symptom severity, functional disability, additional symptoms, and overall health [21]. In China, a Long COVID Symptoms and Severity Score [22], consisting of 44 distinct items with a 4-point scale for severity, and in Taiwan, a Post-COVID-19 Symptom Scale consisting of 24 items with an 11-point scale were both recently validated. While general treatment guidance is evolving and being continually re-evaluated, the lack of consensus regarding Long COVID definitions, the number of symptoms to evaluate, and corresponding agreed-upon models of care persists [23].

In response to the Long-term impact of COVID-19, a validated instrument is essential for advancements in the reporting, evaluation, and treatment of Long COVID in the U.S. The Memorial Symptom Assessment Scale-Long COVID (MSAS-LC) is a new instrument for the assessment of these measures. Symptom data provided by the MSAS-LC expands our understanding of the variability of symptoms, identification of symptom patterns, and homogeneity, and provides a better understanding of those in more urgent need of intervention. This study aimed to assess the prevalence of Long COVID symptoms using the MSAS-LC, evaluate the MSAS-LC in to ease of use, and compare individuals identifying as experiencing symptoms with Long COVID to those without current manifestations.

## 2. Materials and Methods

### 2.1. Instrument Development

Early instruments for assessing the symptoms of Long COVID had more than 200 symptoms. With permission from the creator, Dr. Russell Portenoy, our goal was to develop a briefer tool that covered only the most prevalent symptoms. After a review of many symptom assessment tools, we selected the Memorial Symptom Assessment Scale (MSAS), primarily because of its inclusion of frequency, severity, and distress indicators for each symptom [24]. We revised the tool for use with patients with Long COVID. We defined Long COVID using the WHO criteria requiring the continuation or development of new symptoms after an initial infection, with symptoms lasting more than two months with no alternative explanation [1]. Thus, we revised the time frame in the instructions for the MSAS to specify symptoms lasting longer than three months. We further revised the MSAS by including only the most prevalent symptoms of Long COVID. Those symptoms for the initial version of this modified instrument were determined using a list of the most common items from three articles presenting data on other tools to measure the symptoms of Long COVID at the time [18,21,25]. We included 43% of the 131 symptoms (excluding 13 measurements of quality of life) listed in the SBQ-LC [18]; 70% of the 53 symptoms in The Long COVID Symptom and Impact Tools (ST and IT) [25]; and 100% of the 17 items found in the modified COVID-19 Yorkshire Rehabilitation Scale [21].

We assessed the face validity of our initial pool of symptoms included in the MSAS-LC by asking clinicians (8 physicians and 1 nurse practitioner) who were caring for patients with Long COVID to review and revise the items. They had suggestions for replacing words, e.g., replacing “depression” with “feeling sad, loss of interest”; replacing “urinary symptoms” with “difficulty passing urine or leaking urine”. They asked clarifying questions, e.g., Did runny nose mean runny nose or nasal congestion? Did difficulty sleeping mean falling asleep versus staying asleep? We were asked to consider adding symptoms, such as those found in PTSD, lightheadedness, speech changes, dry mouth, and vertigo; and it was suggested that we delete symptoms, e.g., itchy skin, blurred vision, irritability, weight loss/gain. We carefully considered each suggestion, and when there was disagreement, we adopted the majority consensus from the experts and the literature. In preparation for any participants who may prefer reading the questions in Spanish, all instruments were translated into Spanish by two native-speaking translators, followed by the reconciliation of the translations among translators.

### 2.2. Application of MSAS-LC

#### 2.2.1. Study Design

Administration of the MSAS-LC was conducted using a cross-sectional survey design. The study was approved by Vanderbilt University’s Institutional Review Board (ID# 230164).

#### 2.2.2. Inclusion Criteria

Inclusion criteria for Long COVID participants specified they were over the age of 18; able to speak, read, and write English or Spanish; live in the U.S.; have a device with internet access (smartphone, tablet or computer); have documentation of a positive SARS-CoV-2 diagnostic test or antibody test results, or to be three months from the onset of COVID-19 with symptoms that have lasted for at least two months and cannot be explained by an alternative diagnosis. We limited the sample to those living in the U.S., given that there is significant variation in healthcare systems among countries. We included participants with unconfirmed RT-PCR and antibody cases. This choice is both in line with other studies characterizing Long COVID [26,27], and considers the social, physiological, and environmental barriers that may prevent testing and treatment of COVID-19 for some individuals [28]. Symptoms can independently be a strong indicator of Long COVID, and comparable results in terms of symptom prevalence, trajectory, and disease duration have been reported in comparisons of patients with confirmed and unconfirmed diagnoses [29].

Respondents without Long COVID were screened for eligibility using the same inclusion criteria, with the exclusion of symptoms lasting two months after COVID-19 infection. While many participants in this group had a prior confirmed or suspected COVID-19 infection, a confirmed infection was not required for inclusion. This decision reflects the intent to capture a broader population of individuals without persistent post-COVID symptoms, including those who may not have knowingly had COVID-19. We chose to use individuals without Long COVID to mitigate sampling bias from participants’ self-selection, considering individuals experiencing lasting symptoms may be particularly motivated to participate to gain insights into their own condition or to help others experiencing similar symptoms. Additionally, self-selection may provide a non-representative sample with more symptomatic participants and overestimate the prevalence of persistent symptoms [30].

#### 2.2.3. Recruitment Strategy

Participants were recruited from Vanderbilt University Medical Center’s (VUMC) Adult Post-COVID Clinic; targeted Facebook, Instagram, and Google advertisements; and ResearchMatch, a nonprofit program funded by the National Institutes of Health (NIH) designed to connect eligible participants to Institutional Review Board-approved research studies in the U.S. Study data were collected and managed using Research Electronic Data Capture (REDCap). REDCap is a secure, web-based software platform to support data capture for research studies [31,32]. Per ResearchMatch requirements, email inquiries containing the study objectives and inclusion criteria were sent via ResearchMatch to eligible prospective participants with a link to the REDCap informed consent and participant screening tool. Participants recruited through VUMC and targeted advertisements were prompted to the study’s landing page, which contained an explanation of objectives and eligibility information and a link to the REDCap informed consent and participant screening tool.

#### 2.2.4. Sample Size

We targeted a sample size of 250 participants (125 with LC, 125 without LC) based on the minimal requirement of a total sample size at least 5 times the number of items for relative item response clustering methods. A total of 261 participants (130 with LC, 131 without LC) completed the MSAS-LC and associated questions about the instrument from March–November of 2023. One survey completed by a participant with Long COVID was excluded from the study due to nonsensical responses to write-in survey items, resulting in samples of 129 with LC and 131 without LC for analysis.

#### 2.2.5. Screening and Exclusion

**Figure 1:**
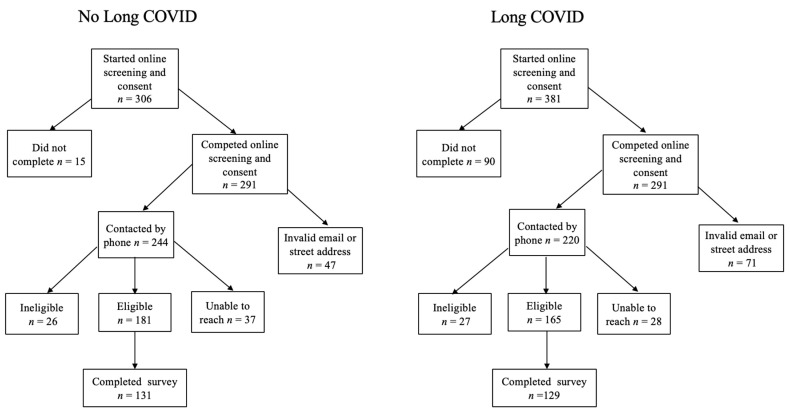
Process of screening and enrollment.

Given the high rate of fraudulent entries for compensated surveys obtained through online recruitment [33], extensive measures were taken to verify prospective participants, including (1) the use of ReCAPTCHA on all tools; (2) email verification through Snov.io, an online tool that verifies email domains as existent and active and checks SMTP authentication; (3) address verification using Google Maps; and (4) scripted phone screenings to match responses to those provided on the questionnaire. (Spanish-speaking telephone screenings were offered and conducted by Spanish-speaking collaborators; this was used by one participant). If prospective participants did not meet the inclusion criteria or fraud was confirmed following the scripted telephone interview, they were informed via email of our reason for exclusion. Our determination process is illustrated in Figure 1.

#### 2.2.6. Data Collection

In addition to completing information about whether or not they had experienced COVID and if they had proof of a positive diagnostic test, participants’ ages, gender, and place of residence were captured using an initial set of questions on the survey. After the stringent screening described above, an individual single-use link to the REDCap surveys was sent to verified email addresses. Each participant completed the MSAS-LC and then completed a set of questions about the instrument. After completion, we conducted additional manual inspections of the surveys to detect duplicate or nonsensical responses to open-ended questions and/or responses to hidden survey items, which are visible to bots but invisible to human respondents. These items were added because online surveys, especially those offering payment, are particularly susceptible to fraud [34]. Personal information, including name, phone number, email, and mailing address provided on the compensation form, was matched to the verified participant before processing payment. Only de-identified data were exported from the REDCap environment for analysis.

#### 2.2.7. Instruments

MSAS-Long COVID (MSAS-LC): The MSAS-Long COVID (MSAS-LC) included 45 symptoms. Instructions at the beginning of the survey prompted participants to carefully consider symptoms they experienced during the past three months. If they did not have the symptom during the past three months, they selected “No” and went on to the next symptom. If the participant selected “Yes”, they were then prompted to indicate how frequently the symptom occurred (1 “Rarely”, 2 “Occasionally”, 3 “Frequently”, or 4 ”Almost Constantly”), the severity of the symptom (1 “Slight”, 2 “Moderate”, 3 “Severe”, or 4 “Very Severe”), and how distressful the symptom was to them (0 “Not at All”, 1 “A Little Bit”, 2 “Somewhat”, 3 “Quite a Bit”, or 4 “Very Much”). Participants could not continue to the next symptom without completing the prior, thus ensuring a complete set of responses for all of the symptoms included in the MSAS-LC. After answering all 45 symptom items, participants were asked to write in any additional symptoms experienced during the past three months that were not included on the MSAS-LC and to indicate how frequently, severe, and distressful the symptom was to them. Responses for frequency, severity, and distress were combined into a global index of burden by summing those responses. Thus, the index of burden for each respective symptom could range from 0 (symptom not experienced) to 12 (a response of 4 for each of the frequency, severity, and distress components).

Questions about the MSAS-LC: Immediately following the completion of the MSAS-LC, participants were asked several questions regarding the difficulty, if any, they experienced in understanding, answering, reading, or choosing responses. If the participant responded “No”, they went to the next question. If the participant responded “Yes”, they were prompted to provide an open-ended response. For the question, “Were the words large enough to read?”, respondents simply selected “No” or “Yes”. They were also given the opportunity to add any symptoms via write-in fields at the end of the MSAS-LC survey.

#### 2.2.8. Data Analysis

We used cross-tabulated frequency distributions to summarize the nominal demographic characteristics of the respondents without and with Long COVID and subsequently compared the groups using Chi-Square tests of independence. Continuous years of age reports were summarized using median and inter-quartile range (IQR), with a Mann–Whitney test being used for the group comparison. Frequency distributions were also used to summarize queries for feedback regarding the MSAS-LC. Each MSAS-LC symptom report (no/yes) was summarized using a cross-tabulated frequency distribution, with the same types of summaries used for each associated frequency, severity, and distress level. We conducted group comparisons of the prevalence observed for each symptom using logistic regression with resulting odds ratios and 95% confidence intervals for the ratio. Symptom burden scores (the combined frequency, severity, and distress responses) were summarized using median and interquartile range (IQR). Given the extremely positively skewed burden score distributions with ‘0’ values (possible range 0–12), we used generalized linear models incorporating the tweedie distribution with a log link to compare the burden scores for each symptom between the study groups. The estimated mean difference (with 95% confidence interval) on the original burden score scale was generated from those models. All interpretations of statistical significance maintained a maximum alpha of 0.05 (*p* < 0.05).

## 3. Results

### 3.1. Demographic Characteristics

Demographic and COVID characteristics of both the groups without (*n* = 131) and with Long COVID (*n* = 128) are summarized in Table 1. Participants with Long COVID were a median of 5 years older than those not reporting Long COVID (*p* = 0.007), with 76.4% (*n* = 85) being 40 years or older (vs. 52.0%, *n* = 68, without Long COVID). Gender groups were approximately equally represented in each group (80.5%, *n* = 103, female with Long COVID, 77.1%, *n* = 101, female without). Almost 60% of those with Long COVID (57.8%, *n* = 74) report onset at least 2 years prior to completing the study survey. Only one participant opted to use the Spanish translation version of the survey.

### 3.2. Data Quality, Face Validity, and Participant Feedback Regarding the MSAS-LC

The survey was programmed in REDCap such that each participant was required to respond to each symptom stem and, if present, required to respond to the subsequent frequency, severity, and distress queries. Thus, all responses for the MSAS-LC were completed by all. Several participants, both without and with Long COVID, wrote in descriptors of additional symptoms, many of which supplemented descriptions of symptoms already in the survey. For example, those with muscle pain identified where the pain was emanating. Generalized fatigue was clarified as morning or intermittent. Changes to taste were listed instead of complete loss, and headaches were further identified as migraines or pressure.

In terms of participant feedback about the MSAS-LC, the highest area of concern was prompted by the question, “Did you have any difficulty answering the questions on the scale?” Overall, 7.7% (*n* = 20 of 259) responded that they did; 10.9% (*n* = 14 of 128) of those with Long COVID responded affirmatively (compared to only 4.7% (*n* = 6) of those without (Table 2).

### 3.3. Prevalence and Burden of MSAS-LC Symptoms in Those Without and with Long COVID

**Figure 2:**
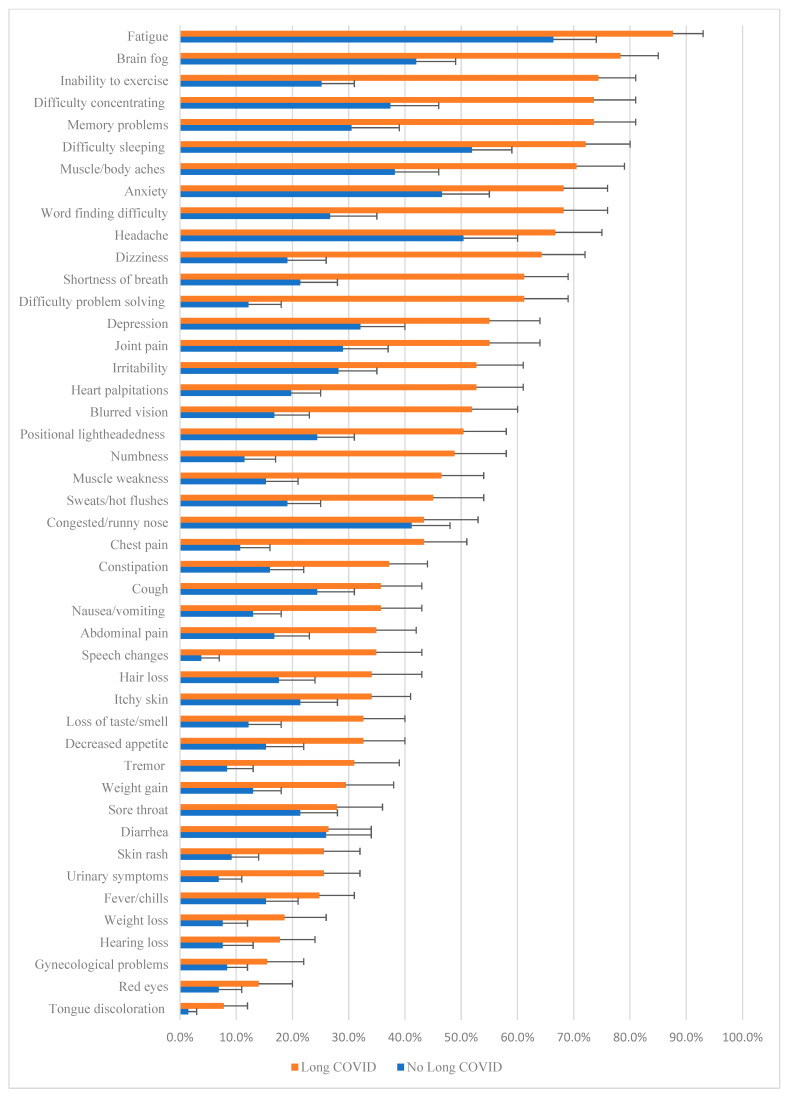
Symptom prevalence in those without and with Long COVID (*n* = 259). Bootstrapped 95% confidence interval error bars.

The prevalence of symptoms reported by participants without and with Long COVID is illustrated in Figure 2. The most commonly identified symptom by both groups was “fatigue” (87.60% and 66.40%, respectively) followed by “brain fog” (78.30%), and “inability to exercise” (74.40%) for those with Long COVID, and “difficulty sleeping” (51.90%) and “headache” (50.4%) for those without Long COVID. There was considerable discrepancy in prevalence for many of the more commonly reported symptoms and a higher prevalence for every symptom for those with Long COVID compared to those without. The least discrepancy in prevalence between groups was for “congested or runny nose” (43.40% and 41.20%, respectively) and “diarrhea” (26.4% and 26.00%, respectively).

We further analyzed our findings regarding prevalence to capture the burden. Then, the symptoms included in the MSAS-LC were organized into general physiological and psychological clusters to assist in identifying shared characteristics, underlying pathophysiology, and affected systems that may reveal underlying mechanisms of the disease. Symptom domain classifications were guided by published literature and are not intended to be definitive. Although symptoms such as fatigue and sleep disturbances are multifactorial in nature, we categorized fatigue under the musculoskeletal domain due to its frequent co-occurrence with muscle pain, joint pain, and post-exertional malaise in Long COVID studies [35,36,37]. We categorized sleep disturbance within the psychiatric domain, consistent with its common association with anxiety, depression, and neuropsychiatric symptoms in Long COVID cohorts [38,39,40].

Given the significant difference in age between the group of participants with Long COVID and those without, for each of the statistical comparisons of burden, we conducted a sensitivity analysis that included age as a covariate in the model. The results were very similar. Where there were any detectable differences, those are noted in the text below and in the respective tables.

### 3.4. Neurologic

Many of the most discriminating symptom experiences reported by the Long COVID participants compared to those without Long COVID were in the neurologic domain. Summaries of the prevalence and associated burden reported for each respective symptom for each group are shown in Table 3, with detailed responses to the burden components summarized in Table S1. Within the pool of symptoms, the most discriminating symptoms between those without and with Long COVID were issues with speech, problem-solving, and memory (*p* < 0.001). Compared to the sample without Long COVID, speech concerns were more than 13 times more likely to be reported by those with Long COVID (34.3% vs. 3.8%, OR = 13.20, 95% CI = 5.03, 34.66), problem-solving more than 11 times more likely (60.9% vs. 12.2%, OR = 11.21, 95% CI = 5.96, 21.10), and memory more than 6 times more likely (73.4% vs. 30.5%, OR = 6.29, 95% CI = 3.76, 10.80).

The symptom burden index had a possible range of 0 (symptom not experienced) to 12 (maximum frequency, severity, and distress). The strongest effects of Long COVID on the burden associated with neurological symptoms were observed for memory, with an estimated mean 4.62 points higher on the 0–12 scale for those experiencing Long COVID compared to those not (95% CI = 3.67, 5.52, *p* < 0.001). Compared to respondents not experiencing Long COVID, those with Long COVID also reported slightly more than a 4-point increase in burden on the 12-point burden scale for symptoms of brain fog and concentration (mean difference = 4.46, 95% CI = 3.48, 5.43, *p* < 0.001 and mean = 4.20, 95% CI = 3.22, 5.17, respectively, *p* < 0.001) (Table 3 and Table S1).

### 3.5. Otolaryngologic

Within the pool of otolaryngologic symptoms, the most discriminating symptoms between those without and with Long COVID were dizziness or lightheadedness, blurred vision, and loss of taste or smell (*p* < 0.001) (Table 4, Table S2). Compared to the sample without Long COVID, dizziness was almost 8 times more likely to be reported by those with Long COVID (OR = 7.56, 95% CI = 4.29, 13.31), blurred vision more than 5 times more likely (OR = 5.27, 95% CI = 2.96, 9.36), and loss of taste or smell more than 3 times more likely (OR = 3.39, 95% CI = 1.78, 6.44).

Similar patterns were observed for the discriminatory value of the burden associated with each of those symptoms. The burden associated with the experience of dizziness and lightheadedness for those with Long COVID was an estimated mean 3.42 points higher on the 0–12 scale than that experienced by participants without Long COVID (95% CI = 2.57, 4.27, *p* < 0.001). Slightly lower increased burden was experienced for those with Long COVID with the symptom of blurred vision (mean = 2.69, 95% CI = 1.86, 3.52, *p* < 0.001) and of loss of taste or smell (mean = 1.86, 95% CI = 1.03, 2.69, *p* < 0.001) (Table 4 and Table S2).

### 3.6. Gastrointestinal

While the prevalence and burden summaries for the gastrointestinal symptoms were generally not as high as those for the most prevalent neurologic and otolaryngologic symptoms, most of the symptoms representing this system on the MSAS-LC differed between the two samples. (Table 5 and Table S3). The largest differences in prevalence between the two groups were for urinary (25.0% vs. 6.9%, OR = 4.52, 95% CI = 2.05, 9.92), nausea (35.2% vs. 13.0%, OR = 3.64, 95% CI = 1.94, 6.80, and constipation symptoms (36.7% vs. 16.0%, OR = 3.04, 95% CI = 1.68, 5.48) (all *p* < 0.001). Within the group with Long COVID, burden values for the symptoms in this domain were generally lower than for symptoms in the neurologic and otolaryngologic domains (all median values = 0, mean differences from those without Long COVID < 2.00) yet remained higher than the respective burden levels for participants without Long COVID for all symptoms except for weight loss and diarrhea (*p* > 0.10) (Table 5 and Table S3).

### 3.7. Psychiatric

Compared to the respondents without Long COVID, the prevalence of symptoms in the psychiatric domain was approximately 2.3 to 2.8 times more likely for those with Long COVID (*p* ≤ 0.001) (Table 6). The largest difference in prevalence was observed for irritability (52.3% vs. 28.2%, OR = 2.79, 95% CI = 1.66, 4.67). The burden associated with sleep symptoms was an estimated mean 2.60 points higher (on a 12-point scale) for those with Long COVID compared to those without (95% C.I. = 1.60, 3.60, *p* < 0.001) (Table 6 and Table S4).

### 3.8. Musculoskeletal

Summaries of the prevalence and associated burden reported for each symptom within the musculoskeletal domain are shown in Table 7, with detailed responses to the burden components summarized in Supplemental Table S5. Within that domain of symptoms, the most discriminating symptoms between those with and without Long COVID were issues with fatigue following (or preventing) exercise (74.2% vs.25.2%, OR = 8.55, 95% CI = 4.88, 14.96) and numbness (48.4% vs.11.5%, OR = 7.27, 95% CI = 3.83, 13.38) (both *p* < 0.001). Furthermore, the burden effect of Long COVID on fatigue related to exercise was an estimated mean 5.13 points higher (on the 0–12-point scale) for those experiencing Long COVID compared to those not (95% CI = 4.17, 6.10, *p* < 0.001) (Table 7 and Table S5).

### 3.9. Unclassified Symptoms

Finally, summaries of the prevalence and associated burden reported for several symptoms that were not classified into a general domain are shown in Table 8 (details in Supplemental Table S6). Among those symptoms, the most discriminating between the participants with and without Long COVID in terms of both prevalence and burden was shortness of breath. Approximately 60% of those with Long COVID reported having the symptom compared to only 21.4% of those without Long COVID (OR = 5.74, 95% CI = 3.31, 9.93) with a burden effect due to shortness of breath an estimated 3.21 points higher with Long COVID than without (95% CI = 2.39, 4.03, *p* < 0.001). No significant differences in prevalence or burden were observed for cough, fever, or chills, and gynecological symptoms (Table 8 and Table S6).

## 4. Discussion

Long COVID participants experienced a range of unresolved symptoms with elevated frequency, severity, and distress compared to individuals without Long COVID. The most common symptoms, fatigue, difficulty sleeping, brain fog, difficulty concentrating, and memory problems, were significantly elevated in every subcategory in comparison to those without. There was considerable discrepancy in prevalence for many of the more commonly reported symptoms and a higher prevalence for every symptom for those with Long COVID compared to those without. These findings highlight the potential public health relevance regarding both individual and collective symptom presentations and burden.

Within the neurological clustering, problem-solving was significant in both prevalence and severity, complementing contemporary neurological prevalence research [41,42]. Equally compelling was the significant variation in prevalence and severity compared to those without Long COVID, suggesting that this specific symptom does not persist and burden the same way in a sample of the general population. Otolaryngologic clustering analysis also illuminated a similar high prevalence of dizziness, lightheadedness, and blurred vision for those with Long COVID, as well as a significant variation in prevalence and severity between those with and without.

A persistent area of concern in Long COVID research is the prevalence of fatigue [43,44]. Our findings reflect the continued prevalence and severity of this symptom and suggest differences in the occurrence and severity in comparison to those without Long COVID, again highlighting the importance of recognizing the validity of the symptoms as an identifier of Long COVID and not merely a general symptom of illness or poor health. Although showing a lower prevalence in our study compared to others [45], respiratory clustering for shortness of breath and chest pain were also divergent in prevalence and severity between groups, further highlighting these symptoms as an area of concern specific to Long COVID.

Our study also provides robust preliminary evidence for the utility of the MSAS-LC.

Data quality for the MSAS-LC was confirmed through stringent screening of online participants and required completion of each survey item. Criterion validity was achieved by incorporating Long COVID experts in the development of the scale, who assessed the face validity of our initial pool of symptoms included in the MSAS-LC. Content validity was assessed in a questionnaire regarding the MSAS-LC, confirming the tool as legible, comprehensive, and easy to use. Given that many of the write-in symptoms supplemented descriptions of symptoms already in the survey, amendments designed to expound and clarify broader descriptors may assist in refining subsequent versions of the MLAS-LC. Longitudinal validation is warranted to assess the MSAS-LC’s ability to monitor symptom changes over time and track treatment response and disease progression. Additionally, future studies should aim to compare the MSAS-LC with other validated Long COVID assessment tools, such as the Yorkshire Rehabilitation Scale, to evaluate convergent validity and clinical utility.

There are limitations to this study. Although self-report measures are valuable for capturing patient experience, they are susceptible to reporting bias. We attempted to mitigate this effect by the inclusion of individuals without COVID. Due to the inherent subjectivity in self-reporting, the results obtained may not entirely reflect the symptom severity. Our sample was also limited to individuals with internet access and the ability to complete an online survey. This may introduce selection bias and limit generalizability, particularly among populations with lower digital literacy or access. Although a Spanish version of the MSAS-LC was available, uptake was minimal. This highlights the need for additional testing and cultural adaptation of the tool across more linguistically and culturally diverse populations. Additionally, we did not collect detailed comorbidity data, which limits our ability to assess how underlying health conditions may have influenced symptom reporting and limits the ability to draw causal conclusions about Long COVID and its unique symptomatology. Although individuals with Long COVID also have pre-existing comorbidities observed in the general population [6], future research should include comorbidity data to better distinguish symptoms uniquely associated with Long COVID. This study also did not collect data on the timing or specific period of infection, limiting our ability to assess whether differences in symptom profiles may be associated with specific viral variants. Future research should incorporate the timing of infection to examine potential relationships between viral strain and long-term symptom presentation. Another limitation of this study is the inexact nature of clustering symptoms by general physiological or psychological characteristics, as many symptoms, such as fatigue and sleep disturbance, may span multiple domains or defy clear categorization. While our classifications were informed by literature, we recognize they are not definitive and may differ across studies.

This study had several strengths, one of which was the use of both participants with Long COVID and without. Without that group, it would be challenging to differentiate disease progression from symptoms originating from other causes [46]. In our fifth year of COVID and subsequent post-COVID conditions, we had substantial research and assessment strategies to build on. The MSAS-LC incorporates years of Long COVID expertise, robust research, and consideration of multiple measurement tools that are now available. Although there is extensive and confirmatory literature regarding prevalence, there was a noticeable dearth of nuanced explorations of severity, particularly into the frequency and distress caused by individual symptoms that the MSAS-LC provides. Additionally, the use of participants without Long COVID provides information on pre-existing symptom prevalence and severity in the population, in which some symptoms might be caused by existing conditions or seasonal illnesses.

To ensure an adequate response to the ongoing Long COVID crisis, we need a measurement tool that builds on existing knowledge and allows those suffering to document their ailments in a complete and individualized way that provides a clearer picture of the personal impact of unrelenting illness. Integrating the MSAS-LC into electronic health records or telehealth platforms could further streamline the healthcare process and enable more targeted treatment strategies based on multidimensional severity categorization. Given the high prevalence and persistence of Long COVID symptoms, it is imperative that the medical community mitigates the burden on individual patients and the collective healthcare system.

## 5. Conclusions

This study introduced a newly developed, promising scale and presents a preliminary validation of a comprehensive, self-administered questionnaire designed to identify and quantify the multifaceted symptomatology of Long COVID. Our findings suggest the potential utility of identifying symptom clusters that differ between individuals with and without Long COVID and may inform future research aimed at characterizing symptom severity and burden. Additionally, the use of participants without Long COVID provides information on pre-existing symptom prevalence and severity in the population, in which some symptoms might be caused by existing conditions or seasonal illnesses. Clustering analysis reveals areas of focus consistent with contemporary research, providing both confirmatory findings and highlighting areas for continuing exploration and focus. The MSAS-LC is a promising tool for the assessment of Long COVID symptoms and may support future efforts to identify and tailor therapies and supportive services. Further validation and longitudinal testing are needed to confirm its clinical applicability.

## Figures and Tables

**Table 1:** Summaries of the demographic and COVID characteristics of participants with and without Long COVID (*n* = 259).

	Demographic Data and Characteristics		
	No Long COVID (*n* = 131)	Long COVID (*n* = 128)	*p* Value *
Age (years, median, IQR)	41 (32, 54)	46 (36, 58)	0.007
	*n* (%)	*n* (%)	
Age group (years)			0.06
18–39	63 (48.0)	43 (33.6)	
40–59	45 (34.4)	57 (44.5)	
≥60	23 (17.6)	28 (21.9)	
Gender			0.51
Female	101 (77.1)	103 (80.5)	
Male	30 (22.9)	25 (19.5)	
Prior COVID			NA
No	36 (27.5)	0 (0.0)	
Yes	95 (72.5)	128 (100.0)	
Of those reporting prior COVID, proof of a COVID Diagnosis	N = 95		<0.001
No	45 (47.4)	23 (18.0)	
Yes	50 (52.6)	105 (82.0)	
Onset of Long COVID symptoms			
<1 year	-----	6 (4.7)	
≥1 year	-----	48 (37.5)	
≥2 years	-----	29 (22.6)	
≥3 years	-----	45 (35.2)	

* Mann–Whitney test (age years); Pearson Chi-Square test (all other categorical variables). Abbreviation: IQR = interquartile range.

**Table 2:** Summaries of questions asked regarding the MSAS-LC.

	Long COVID (*n* = 128)		No Long COVID (*n* = 131)		Total (*n* = 259)	
Survey Question	No *n* (%)	Yes *n* (%)	No *n* (%)	Yes *n* (%)	No *n* (%)	Yes *n* (%)
1. Did you have any difficulty understanding the instructions on the scale?	121 (94.5)	7 (5.5)	129 (98.4)	2 (1.6)	250 (96.5)	9 (3.5)
2. Did you have any difficulty answering the questions on the scale?	114 (89.1)	14 (10.9)	125 (95.3)	6 (4.7)	239 (92.2)	20 (7.8)
3. Were the words large enough to read?	3 (2.3)	125 (97.7)	8 (6.2)	123 (93.8)	11 (4.3)	248 (95.7)
4. There were 3 questions we asked about each symptom: how often you have it, how severe it was, and how much distress or bother it caused you. Did you have any difficulty understanding these aspects of the symptoms?	122 (95.3)	6 (4.7)	131 (100)	0 (0)	253 (97.7)	6 (2.3)
5. Did you have any difficulty understanding the different choices you had for each of the 3 questions about each symptom?	119 (93)	9 (7.0)	131 (100)	0 (0.0)	250 (96.5)	9 (3.5)
6. Were there any symptom words that you did not understand?	122 (95.3)	6 (4.7)	129 (98.4)	2 (1.6)	251 (96.9)	8 (3.1)
7. You had room to add up to 7 additional symptoms that were not listed on the scale. Did you need more than 7 spaces for symptoms? If so, about how many more?	128 (100.0)	0 (0.0)	131 (100.0)	0 (0.0)	259 (100.0)	0 (0.0)

**Table 3:** Summaries of the prevalence and burden associated with neurological symptoms in those without and with Long COVID (*n* = 259).

	Prevalence				Burden *			
	Long COVID				Long COVID			
	No (*n* = 131) *n* %	Yes (*n* = 128) *n* %	*p* Value **	OR (95% C.I.)	No (*n* = 131) Median (IQR) Min, Max	Yes (*n* = 128) Median (IQR) Min, Max	*p* Value ***	Estimated Mean Difference ^a^ (95% C.I.)
Memory	40 (30.5)	94 (73.4)	<0.001	6.29 (3.66, 10.80)	0.0 (0.0, 4.0) *0, 11*	7.0 (0.0, 10.8) *0, 12*	<0.001	4.60 (3.67, 5.52)
Brain fog	55 (42.0)	100 (78.1)	<0.001	4.94 (2.86, 8.51)	0.0 (0.0, 6.0) *0, 12*	9.0 (4.0, 11.0) *0, 12*	<0.001	4.46 (3.48, 5.43)
Concentration	49 (37.4)	94 (73.4)	<0.001	4.63 (2.72, 7.85)	0.0 (0.0, 5.0) *0, 12*	8.0 (0.0, 11.0) *0, 12*	<0.001	4.20 (3.22, 5.17)
Word finding	35 (26.7)	87 (68.0)	<0.001	5.82 (3.40, 9.96)	0.0 (0.0, 3.0) *0, 10*	6.0 (0.0, 10.0) *0, 12*	<0.001	3.96 (3.03, 4.89)
Problem solving	16 (12.2)	78 (60.9)	<0.001	11.21 (5.96, 21.10)	0.0 (0.0, 0.0) *0, 12*	5.0 (0.0, 9.0) *0, 12*	<0.001	3.89 (3.03, 4.74)
Speech	5 (3.8)	44 (34.4)	<0.001	13.20 (5.03, 34.66)	0.0 (0.0, 0.0) *0, 9*	0.0 (0.0, 6.0) *0, 12*	<0.001	2.44 (1.72, 3.15)
Headache	66 (50.4)	85 (66.4)	0.009	1.95 (1.17, 3.22)	3.0 (0.0, 6.0) *0, 10*	6.0 (0.0, 8.8) *0, 12*	<0.001	1.99 (1.08, 2.90)
Tremor	11 (8.4)	39 (30.5)	<0.001	4.78 (2.32, 9.86)	0.0 (0.0, 0.0) *0, 12*	0.0 (0.0, 6.0) *0, 11*	<0.001	1.69 (0.98, 2.39)

Abbreviations: OR = odds ratio; C.I. = confidence interval; IQR = interquartile range; min = minimum; max = maximum. ^a^ Generalized linear model, tweedie distribution with log link; estimated mean differences were estimated from that model and reported on the 0–12 scale of the global scores. * Global score of symptom burden created by summing the responses to the frequency (1–4), severity (1–4), and distress (1–4); possible range of scores 0 ‘Symptom not experienced’ to 12. ** Logistic regression. *** Generalized linear model, tweedie distribution with log link; estimated mean differences were estimated from that model and reported on the 0–12 scale of the global scores.

**Table 4:** Summaries of the prevalence and burden associated with otolaryngologic symptoms in those without and with Long COVID (*n* = 259).

	Prevalence				Burden *			
	Long COVID				Long COVID			
	No (*n* = 131) *n* %	Yes (*n* = 128) *n* %	*p* Value **	OR (95% C.I.)	No (*n* = 131) Median (IQR) Min, Max	Yes (*n* = 128) Median (IQR) Min, Max	*p* Value ***	Estimated Mean Difference ^a^ (95% C.I.)
Dizziness/lightheadedness	25 (19.1)	82 (64.1)	<0.001	7.56 (4.29, 13.31)	0.0 (0.0, 0.0) 0, 11	5.0 (0.0, 8.0) 0, 12	<0.001	3.42 (2.57, 4.27)
Blurred vision	22 (16.8)	66 (51.6)	<0.001	5.27 (2.96, 9.37)	0.0 (0.0, 0.0) 0, 10	5.0 (0.0, 8.0) 0, 12	<0.001	2.69 (1.86, 3.52)
Loss of taste or smell	16 (12.2)	41 (32.0)	<0.001	3.39 (1.78, 6.44)	0.0 (0.0, 0.0) 0, 12	5.0 (0.0, 8.0) 0, 12	<0.001	1.86 (1.03, 2.69)
Hearing loss	10 (7.6)	22 (17.2)	0.02 ^b^	2.51 (1.13, 5.55)	0.0 (0.0, 0.0) 0, 10	5.0 (0.0, 8.0) 0, 12	0.02	0.76 (0.14, 1.39)
Tongue discoloration/enlargement	2 (1.5)	9 (7.0)	0.05	4.88 (1.03, 23.04)	0.0 (0.0, 0.0) 0, 11	5.0 (0.0, 8.0) 0, 6	0.03	0.41 (0.09, 0.73)
Red eyes	9 (6.9)	17 (13.3)	0.09	2.08 (0.88, 4.85)	0.0 (0.0, 0.0) 0, 9	5.0 (0.0, 8.0) 0, 12	0.08	0.49 (−0.02, 1.01)
Sore throat	28 (21.4)	35 (27.3)	0.26	1.38 (0.78, 2.45)	0.0 (0.0, 0.0) 0, 8	5.0 (0.0, 8.0) 0, 12	0.18	0.58 (−0.03, 1.19)
Congestion/runny nose	54 (41.2)	55 (43.0)	0.78	1.07 (0.65, 1.76)	0.0 (0.0, 5.0) 0, 9	5.0 (0.0, 8.0) 0, 12	0.28	0.61 (−0.21, 1.43)

Abbreviations: OR = odds ratio; C.I. = confidence interval; IQR = interquartile range; min = minimum; max = maximum. ^a^ Generalized linear model, tweedie distribution with log link; estimated mean differences were estimated from that model and reported on the 0–12 scale of the global scores. * Global score of symptom burden created by summing the responses to the frequency (1–4), severity (1–4), and distress (1–4); possible range of scores 0 ‘Symptom not experienced’ to 12. ** Logistic regression *** Generalized linear model, tweedie distribution with log link; estimated mean differences were estimated from that model and reported on the 0–12 scale of the global scores. ^b^ Covaried for age: *p* = 0.05, OR = 2.21, 95% CI = 0.98, 4.94.

**Table 5:** Summaries of the prevalence and burden associated with gastrointestinal symptoms in those without and with Long COVID (*n* = 259).

	Prevalence				Burden *			
	Long COVID				Long COVID			
	No (*n* = 131) *n* %	Yes (*n* = 128) *n* %	*p* Value **	OR (95% C.I.)	No (*n* = 131) Median (IQR) Min, Max	Yes (*n* = 128) Median (IQR) Min, Max	*p* Value ***	Estimated Mean Difference ^a^ (95% C.I.)
Constipation	21 (16.0)	47 (36.7)	<0.001	3.04 (1.68, 5.48)	0.0 (0.0, 0.0) 0, 9	0.0 (0.0, 6.0) 0, 12	<0.001	1.70 (0.90, 2.49)
Urinary	9 (6.9)	32 (25.0)	<0.001	4.52 (2.05, 9.92)	0.0 (0.0, 0.0) 0, 11	0.0 (0.0, 3.0) 0, 12	<0.001	1.40 (0.70, 2.09)
Nausea	17 (13.0)	45 (35.2)	<0.001	3.64 (1.94, 6.80)	0.0 (0.0, 0.0) 0, 11	0.0 (0.0, 4.0) 0, 12	<0.001	1.38 (0.73, 2.04)
Weight gain	17 (13.0)	38 (29.7)	0.001	2.83 (1.50, 5.35)	0.0 (0.0, 0.0) 0, 9	0.0 (0.0, 5.0) 0, 12	<0.001	1.62 (0.84, 2.40)
Decreased appetite	20 (15.3)	41 (32.0)	0.002	2.62 (1.43, 4.79)	0.0 (0.0, 0.0) 0, 8	0.0 (0.0, 5.0) 0, 12	<0.001	1.17 (0.53, 1.80)
Abdominal pain	22 (16.8)	44 (34.4)	0.001	2.60 (1.44, 4.67)	0.0 (0.0, 0.0) 0, 10	0.0 (0.0, 5.0) 0, 12	0.003	1.08 (0.31, 1.86)
Weight loss	10 (7.6)	23 (18.0)	0.02	2.65 (1.20, 5.83)	0.0 (0.0, 0.0) 0, 10	0.0 (0.0, 0.0) 0, 9	0.13	0.37 (−0.11, 0.85)
Diarrhea	34 (26.0)	33 (25.8)	0.98	0.99 (0.56, 1.73)	0.0 (0.0, 3.0) 0, 9	0.0 (0.0, 3.0) 0, 12	0.97	0.08 (−0.63, 0.78)

Abbreviations: OR = odds ratio; C.I. = confidence interval; IQR = interquartile range; min = minimum; max = maximum. ^a^ Generalized linear model, tweedie distribution with log link; estimated mean differences were estimated from that model and reported on the 0–12 scale of the global scores. * Global score of symptom burden created by summing the responses to the frequency (1–4), severity (1–4), and distress (1–4); possible range of scores 0 ‘Symptom not experienced’ to 12. ** Logistic regression *** Generalized linear model, tweedie distribution with log link; estimated mean differences were estimated from that model and reported on the 0–12 scale of the global scores.

**Table 6:** Summaries of the prevalence and burden of psychiatric and cardiovascular symptoms in those without and with Long COVID *n* = 259).

	Prevalence				Burden *			
	Long COVID				Long COVID			
	No (*n* = 131) *n* %	Yes (*n* = 128) *n* %	*p* Value **	OR (95% C.I.)	No (*n* = 131) Median (IQR) Min, Max	Yes (*n* = 128) Median (IQR) Min, Max	*p* Value ***	Estimated Mean Difference ^a^ (95% C.I.)
Psychiatric								
Sleep	68 (51.9)	92 (71.9)	0.001	2.37 (1.41, 3.97)	3.0 (0.0, 7.0) 0, 12	7.0 (0.0, 10.0) 0, 12	<0.001	2.60 (1.60, 3.60)
Anxiety	61 (46.6)	87 (68.0)	<0.001	2.44 (1.47, 4.04)	0.0 (0.0, 7.0) 0, 12	6.0 (4.0, 9.0) 0, 12	<0.001	1.97 (1.01, 2.94)
Depression	42 (32.1)	70 (54.7)	<0.001	2.56 (1.54, 4.25)	0.0 (0.0, 6.0) 0, 12	4.0 (0.0, 8.0) 0, 12	<0.001	1.95 (0.98, 2.91)
Irritability	37 (28.2)	67 (52.3)	<0.001	2.79 (1.66, 4.67)	0.0 (0.0, 4.0) 0, 12	3.0 (0.0, 7.0) 0, 12	<0.001	1.87 (0.98, 2.76)
Cardiovascular								
Chest pain	14 (10.7)	55 (43.0)	<0.001	6.30 (3.26, 12.13)	0.0 (0.0, 0.0) 0, 11	0.0 (0.0, 6.0) 0, 12	<0.001	2.13 (1.41, 2.86)
Heart palpations	26 (19.8)	87 (52.3)	0.009	4.44 (2.55, 7.71)	0.0 (0.0, 0.0) 0, 11	3.0 (0.0, 7.0) 0, 12	<0.001	2.67 (1.85, 3.48)
Vertigo	32 (24.4)	64 (50.0)	<0.001	3.09 (1.82, 5.25)	0.0 (0.0, 0.0) 0, 11	1.5 (0.0, 8.0) 0, 12	<0.001	2.27 (1.39, 3.15)

Abbreviations: OR = odds ratio; C.I. = confidence interval; IQR = interquartile range; min = minimum; max = maximum. ^a^ Generalized linear model, tweedie distribution with log link; estimated mean differences were estimated from that model and reported on the 0–12 scale of the global scores. * Global score of symptom burden created by summing the responses to the frequency (1–4), severity (1–4), and distress (1–4); possible range of scores 0 ‘Symptom not experienced’ to 12. ** Logistic regression *** Generalized linear model, tweedie distribution with log link; estimated mean differences were estimated from that model and reported on the 0–12 scale of the global scores.

**Table 7:** Summaries of the prevalence and burden of musculoskeletal symptoms in those without and with Long COVID *n* = 259).

	Prevalence				Burden *			
	Long COVID				Long COVID			
	No (*n* = 131) *n* %	Yes (*n* = 128) *n* %	*p* Value **	OR (95% C.I.)	No (*n* = 131) Median (IQR) Min, Max	Yes (*n* = 128) Median (IQR) Min, Max	*p* Value ***	Estimated Mean Difference ^a^ (95% C.I.)
Fatigue	87 (66.4)	112 (87.5)	<0.001	3.54 (1.87, 6.70)	5.0 (0.0, 7.0) 0, 11	9.0 (6.2, 11.0) 0, 12	<0.001	3.58 (2.71, 4.45)
Fatigue (exercise)	33 (25.2)	95 (74.2)	<0.001	8.55 (4.88, 14.96)	0.0 (0.0, 4.0) 0, 12	8.0 (0.0, 11.0) 0, 12	<0.001	5.13 (4.17, 6.10)
Muscle aches	50 (38.2)	90 (70.3)	<0.001	3.84 (2.28, 6.44)	0.0 (0.0, 5.0) 0, 11	7.0 (0.0, 9.0) 0, 12	<0.001	3.22 (2.31, 4.13)
Joint pain	38 (29.0)	70 (54.7)	<0.001	2.95 (1.76, 4.94)	0.0 (0.0, 5.0) 0, 12	5.0 (0.0, 8.0) 0, 12	<0.001	2.32 (1.37, 3.27)
Numbness	15 (11.5)	62 (48.4)	<0.001	7.27 (3.83, 13.78)	0.0 (0.0, 0.0) 0, 10	0.0 (0.0, 8.0) 0, 12	<0.001	2.90 (2.06, 3.74)
Muscle weakness	20 (15.3)	59 (46.1)	0.009	4.75 (2.63, 8.56)	0.0 (0.0, 0.0) 0, 11	0.0 (0.0, 8.0) 0, 12	<0.001	2.91 (2.04, 3.79)

Abbreviations: OR = odds ratio; C.I. = confidence interval; IQR = interquartile range; min = minimum; max = maximum. ^a^ Generalized linear model, tweedie distribution with log link; estimated mean differences were estimated from that model and reported on the 0–12 scale of the global scores. * Global score of symptom burden created by summing the responses to the frequency (1–4), severity (1–4), and distress (1–4); possible range of scores 0 ‘Symptom not experienced’ to 12. ** Logistic regression *** Generalized linear model, tweedie distribution with log link; estimated mean differences were estimated from that model and reported on the 0–12 scale of the global scores.

**Table 8:** Summaries of the prevalence and burden of unclassified symptoms in those without and with Long COVID *n* = 259).

	Prevalence				Burden *			
	Long COVID				Long COVID			
	No (*n* = 131) *n* %	Yes (*n* = 128) *n* %	*p* Value **	OR (95% C.I.)	No (*n* = 131) Median (IQR) Min, Max	Yes (*n* = 128) Median (IQR) Min, Max	*p* Value ***	Estimated Mean Difference ^a^ (95% C.I.)
Shortness of breath	28 (21.4)	78 (60.9)	<0.001	5.74 (3.31, 9.93)	0.0 (0.0, 0.0) 0, 11	5.0 (0.0, 8.0) 0, 12	<0.001	3.21 (2.39, 4.03)
Sweats/hot flushes	25 (19.1)	57 (44.5)	<0.001	3.40 (1.94, 5.95)	0.0 (0.0, 0.0) 0, 12	0.0 (0.0, 6.0) 0, 11	<0.001	1.69 (0.92, 2.47)
Cough	32 (24.4)	45 (35.2)	0.06	1.68 (0.98, 2.88)	0.0 (0.0, 0.0) 0, 10	0.0 (0.0, 5.0) 0, 12	0.04 ^b^	0.82 (0.08, 1.56)
Hair loss	23 (17.6)	43 (33.6)	0.003	2.38 (1.32, 4.25)	0.0 (0.0, 0.0) 0, 11	0.0 (0.0, 5.8) 0, 12	0.002	1.36 (0.50, 2.21)
Itchy skin	28 (21.4)	43 (33.6)	0.03	1.86 (1.06, 3.25)	0.0 (0.0, 0.0) 0, 9	0.0 (0.0, 4.0) 0, 12	0.02	0.91 (0.18, 1.65)
Skin rash	12 (9.2)	32 (25.0)	0.001	3.31 (1.61, 6.77)	0.0 (0.0, 0.0) 0, 8	0.0 (0.0, 2.3) 0, 12	<0.001	1.30 (0.67, 1.94)
Fever/chills	20 (15.3)	31 (24.2)	0.07	1.77 (0.95, 3.32)	0.0 (0.0, 0.0) 0, 9	0.0 (0.0, 0.0) 0, 11	0.05	0.61 (0.03, 1.20)
Gynecological	11 (8.4)	20 (15.6)	0.08	2.02 (0.92, 4.41)	0.0 (0.0, 0.0) 0, 11	0.0 (0.0, 0.0) 0, 12	0.09	0.51 (−0.09, 1.11)

Abbreviations: OR = odds ratio; C.I. = confidence interval; IQR = interquartile range; min = minimum; max = maximum. ^a^ Generalized linear model, tweedie distribution with log link; estimated mean differences were estimated from that model and reported on the 0–12 scale of the global scores. * Global score of symptom burden created by summing the responses to the frequency (1–4), severity (1–4), and distress (1–4); possible range of scores 0 ‘Symptom not experienced’ to 12. ** Logistic regression *** Generalized linear model, tweedie distribution with log link; estimated mean differences were estimated from that model and reported on the 0–12 scale of the global scores. ^b^ Covaried for age: *p* = 0.06, Mean difference = 0.69, 95% CI = −0.02, 1.40.

## Data Availability

Datasets used and/or analyzed during the current study are available from the corresponding author upon request.

## Supplementary Materials

The following supporting information can be downloaded at https://www.mdpi.com/article/10.3390/ijerph22050728/s1. Table S1: Within the samples reporting specific neurological symptoms, summaries of the respective frequency, severity, and bother responses for participants without and with Long COVID; Table S2. Within the samples reporting specific otolaryngologic symptoms, summaries of the respective frequency, severity, and bother responses for participants without and with Long COVID; Table S3. Within the samples reporting specific gastrointestinal symptoms, summaries of the respective frequency, severity, and bother responses for participants without and with Long COVID; Table S4. Within the samples reporting specific psychiatric and cardiovascular symptoms, summaries of the respective frequency, severity, and bother responses for participants without and with Long COVID; Table S5. Within the samples reporting specific musculoskeletal symptoms, summaries of the respective frequency, severity, and bother responses for participants without and with Long COVID; Table S6. Within the samples reporting specific unclassified symptoms, summaries of the respective frequency, severity, and bother responses for participants without and with Long COVID.

## Abbreviations

The following abbreviations are used in this manuscript:

MSAS-LC	Memorial Symptom Assessment Scale-Long COVID
WHO	World Health Organization
SBQ-LC	The Symptom Burden Questionnaire for Long COVID
PTSD	Post Traumatic Stress Disorder
RT-PCR	Reverse transcription polymerase chain reaction
REDCap	Research Electronic Data Capture
ReCAPTCHA	Revised Completely Automated Public Turing test to Tell Computers and Humans Apart
