# Supplementary material for: The Development and Initial Validation of the Memorial Symptom Assessment Scale-Long COVID (MSAS-LC): A Promising Tool for Measuring Long COVID
Source: Int J Environ Res Public Health. 2025 May 2;22(5):728. doi: 10.3390/ijerph22050728 (PMC12110800; doi:10.3390/ijerph22050728)
Supplement: Supplementary file 1 [file ijerph-22-00728-s001.zip › ijerph-3522771-supplementary.pdf]

Table S1. Within the samples reporting specific neurological symptoms, summaries of the respective frequency, severity, and bother responses for participants without and with long COVID.

	Without Long COVID (N = 131)					*With Long COVID (N = 128)*			
	N	Frequency	Severity	Bother		N	Frequency	Severity	Bother
		Frequently/ Constantly	Moderate/ Severe	Quite a Bit/ Very Much			Frequently/ Constantly	Moderate/ Severe	Quite a Bit / Very Much
		n (%)	n (%)	n (%)			n (%)	n (%)	n (%)
Memory	40	12 (30.0)	6 (15.0)	11 (27.5)		94	69 (73.4)	44 (46.8)	70 (74.5)
Brain fog	55	22 (40.0)	12 (21.8)	23 (41.8)		100	79 (79.0)	59 (59.0)	82 (82.0)
Concentration	49	21 (42.9)	10 (20.4)	16 (32.7)		94	78 (83.0)	52 (55.3)	66 (70.2)
Word finding	35	15 (42.9)	9 (25.7)	14 (40.0)		87	59 (67.8)	37 (42.5)	60 (69.0)
Problem solving	16	5 (31.3)	5 (31.3)	9 (56.3)		78	45 (57.7)	30 (38.5)	48 (61.5)
Speech	5	2 (40.0)	1 (20.0)	2 (40.0)		44	24 (54.5)	17 (38.6)	29 (65.9)
Headache	66	23 (34.8)	16 (24.2)	18 (27.3)		85	51 (60.0)	37 (43.5)	41 (48.2)
Tremor	11	5 (45.5)	2 (18.2)	3 (27.3)		39	24 (61.5)	13 (33.3)	19 (48.7)

Table S2. Within the samples reporting specific otolaryngologic symptoms, summaries of the respective frequency, severity, and bother responses for participants without and with long COVID.

	Without Long COVID (N = 131)					*With Long COVID (N = 128)*			
	N	Frequency	Severity	Bother		N	Frequency	Severity	Bother
		Frequently/ Constantly	Moderate/ Severe	Quite a Bit/ Very Much			Frequently/ Constantly	Moderate/ Severe	Quite a Bit / Very Much
		n (%)	n (%)	n (%)			n (%)	n (%)	n (%)
Dizziness / lightheadedness	25	13 (52.0)	9 (36.0)	10 (40.0)		82	43 (52.4)	25 (30.5)	42 (51.2)
Blurred vision	22	11 (50.0)	5 (22.7)	6 (27.3)		66	35 (53.0)	16 (24.2)	36 (54.5)
Loss of taste or smell	16	4 (25.0)	7 (43.8)	7 (43.8)		41	27 (65.9)	22 (53.7)	21 (51.2)
Hearing loss	10	7 (70.0)	1 (10.0)	4 (40.0)		22	14 (63.6)	5 (22.7)	12 (54.5)
Tongue discoloration / enlargement	2	0 (0.0)	0 (0.0)	1 (11.1)		9	7 (77.8)	1 (11.1)	1 (11.1)
Red eyes	9	4 (44.4)	2 (22.2)	3 (33.3)		17	12 (70.6)	3 (17.6)	6 (35.3)
Sore throat	28	2 (7.1)	2 (7.1)	2 (7.1)		35	13 (37.1)	5 (14.3)	8 (22.9)
Congestion / runny nose	54	17 (31.5)	6 (11.1)	8 (14.8)		55	41 (74.5)	10 (18.2)	16 (29.1)

Table S3. Within the samples reporting specific gastrointestinal symptoms, summaries of the respective frequency, severity, and bother responses for participants without and with long COVID.

	Without Long COVID (N = 131)					*With Long COVID (N = 128)*			
	N	Frequency	Severity	Bother		N	Frequency	Severity	Bother
		Frequently/ Constantly	Moderate/ Severe	Quite a Bit/ Very Much			Frequently/ Constantly	Moderate/ Severe	Quite a Bit / Very Much
		n (%)	n (%)	n (%)			n (%)	n (%)	n (%)
Constipation	21	9 (42.9)	7 (33.3)	7 (33.3)		47	25 (53.2)	13 (27.7)	24 (51.1)
Urinary	9	7 (77.8)	3 (33.3)	6 (66.7)		32	22 (68.8)	10 (31.3)	18 (56.3)
Nausea	17	3 (17.6)	5 (29.4)	7 (41.2)		45	16 (35.6)	7 (15.6)	13 (28.9)
Weight gain	17	6 (35.3)	3 (17.6)	8 (47.1)		38	23 (60.5)	13 (34.2)	30 (78.9)
Decreased appetite	20	10 (50.0)	1 (5.0)	1 (5.0)		41	25 (61.0)	10 (24.4)	4 (9.8)
Abdominal pain	22	13 (59.1)	10 (45.5)	11 (50.0)		44	17 (38.6)	14 (31.8)	13 (29.5)
Weight loss	10	6 (60.0)	3 (30.0)	4 (40.0)		23	7 (30.4)	3 (13.0)	1 (4.3)
Diarrhea	34	12 (35.3)	8 (23.5)	8 (23.5)		33	10 (30.3)	6 (18.2)	14 (42.4)

Table S4. Within the samples reporting specific psychiatric and cardiovascular symptoms, summaries of the respective frequency, severity, and bother responses for participants without and with long COVID

	Without Long COVID (N = 131)					*With Long COVID (N = 128)*			
	N	Frequency	Severity	Bother		N	Frequency	Severity	Bother
		Frequently/ Constantly	Moderate/ Severe	Quite a Bit/ Very Much			Frequently/ Constantly	Moderate/ Severe	Quite a Bit / Very Much
		n (%)	n (%)	n (%)			n (%)	n (%)	n (%)
Psychiatric									
Sleep	68	37 (54.4)	18 (26.5)	27 (39.7)		92	71 (77.2)	49 (53.3)	57 (62.0)
Anxiety	61	31 (50.8)	18 (29.5)	24 (39.3)		87	53 (60.9)	35 (40.2)	44 (50.6)
Depression	42	25 (59.5)	13 (31.0)	22 (52.4)		70	46 (65.7)	29 (41.4)	35 (50.0)
Irritability	37	15 (40.5)	7 (18.9)	15 (40.5)		67	38 (56.7)	20 (29.9)	31 (46.3)
Cardiovascular									
Vertigo	32	13 (40.6)	9 (28.1)	10 (31.3)		64	36 (56.3)	24 (37.5)	35 (54.7)
Heart palpations	26	6 (23.1)	5 (19.2)	6 (23.1)		67	45 (67.2)	16 (23.9)	31 (46.3)
Chest pain	14	4 (28.6)	6 (42.9)	5 (35.7)		55	22 (40.0)	12 (21.8)	17 (30.9)

Table S5. Within the samples reporting specific musculoskeletal symptoms, summaries of the respective frequency, severity, and bother responses for participants without and with long COVID

	Without Long COVID (N = 131)					*With Long COVID (N = 128)*			
	N	Frequency	Severity	Bother		N	Frequency	Severity	Bother
		Frequently/ Constantly	Moderate/ Severe	Quite a Bit/ Very Much			Frequently/ Constantly	Moderate/ Severe	Quite a Bit / Very Much
		n (%)	n (%)	n (%)			n (%)	n (%)	n (%)
Fatigue	87	44 (50.6)	21 (24.1)	34 (39.1)		112	98 (87.5)	67 (59.8)	82 (73.2)
Fatigue (exercise)	33	16 (48.5)	10 (30.3)	12 (36.4)		95	76 (80.0)	60 (63.2)	71 (74.7)
Muscle aches	50	22 (44.0)	8 (16.0)	16 (32.0)		90	66 (73.3)	33 (36.7)	52 (57.8)
Joint pain	38	23 (60.5)	11 (28.9)	10 (26.3)		70	50 (71.4)	28 (40.0)	38 (54.3)
Numbness	15	10 (66.7)	5 (33.3)	7 (46.7)		62	42 (67.7)	24 (38.7)	29 (46.8)
Muscle weakness	20	7 (35.0)	5 (25.0)	6 (30.0)		59	45 (76.3)	24 (40.7)	40 (67.8)

Table S6. Within the samples reporting specific unclassified symptoms, summaries of the respective frequency, severity, and bother responses for participants without and with long COVID

	Without Long COVID (N = 131)					*With Long COVID (N = 128)*			
	N	Frequency	Severity	Bother		N	Frequency	Severity	Bother
		Frequently/ Constantly	Moderate/ Severe	Quite a Bit/ Very Much			Frequently/ Constantly	Moderate/ Severe	Quite a Bit / Very Much
		n (%)	n (%)	n (%)			n (%)	n (%)	n (%)
Shortness of breath	28	4 (14.3)	3 (10.7)	8 (18.6)		78	37 (47.4)	21 (26.9)	42 (53.8)
Sweats / hot flushes	25	11 (44.0)	6 (24.0)	10 (40.0)		57	27 (47.4)	12 (21.1)	19 (33.3)
Cough	32	15 (46.9)	6 (18.8)	6 (18.8)		45	22 (48.9)	8 (17.8)	11 (24.4)
Hair loss	23	14 (60.9)	6 (26.1)	16 (69.6)		43	27 (62.8)	13 (30.2)	26 (60.5)
Itchy skin	28	15 (53.6)	2 (7.1)	4 (14.3)		43	18 (41.9)	12 (27.9)	14 (32.6)
Skin rash	12	3 (25.0)	1 (8.3)	1 (8.3)		32	18 (56.3)	7 (21.9)	12 (37.5)
Fever / chills	20	3 (15.0)	3 (15.0)	5 (25.0)		31	11 (35.5)	2 (6.5)	6 (19.4)
Gynecological	11	4 (36.4)	4 (35.4)	5 (45.5)		20	12 (60.0)	8 (40.0)	11 (55.0)
